# Analysis of the Genes Involved in Thiocyanate Oxidation during Growth in Continuous Culture of the Haloalkaliphilic Sulfur-Oxidizing Bacterium *Thioalkalivibrio thiocyanoxidans* ARh 2^T^ Using Transcriptomics

**DOI:** 10.1128/mSystems.00102-17

**Published:** 2017-12-26

**Authors:** Tom Berben, Cherel Balkema, Dimitry Y. Sorokin, Gerard Muyzer

**Affiliations:** a Microbial Systems Ecology, Department of Freshwater and Marine Ecology, Institute for Biodiversity and Ecosystem Dynamics, University of Amsterdam, Amsterdam, The Netherlands; b Winogradsky Institute of Microbiology, Research Centre of Bioengineering, RAS, Moscow, Russian Federation; c Department of Biotechnology, Delft University of Technology, Delft, The Netherlands; Institute for Genomics & Systems Biology

**Keywords:** chemolithoautotrophs, chemostat, RNA-Seq, soda lakes, *Thioalkalivibrio*, thiocyanate, thiocyanate dehydrogenase

## Abstract

Thiocyanate (N=C−S^−^) is a moderately toxic, inorganic sulfur compound. It occurs naturally as a by-product of the degradation of glucosinolate-containing plants and is produced industrially in a number of mining processes. Currently, two pathways for the primary degradation of thiocyanate in bacteria are recognized, the carbonyl sulfide pathway and the cyanate pathway, of which only the former has been fully characterized. Use of the cyanate pathway has been shown in only 10 strains of *Thioalkalivibrio*, a genus of obligately haloalkaliphilic sulfur-oxidizing *Gammaproteobacteria* found in soda lakes. So far, only the key enzyme in this reaction, thiocyanate dehydrogenase (TcDH), has been purified and studied. To gain a better understanding of the other genes involved in the cyanate pathway, we conducted a transcriptomics experiment comparing gene expression during the growth of *Thioalkalivibrio thiocyanoxidans* ARh 2^T^ with thiosulfate with that during its growth with thiocyanate. Triplicate cultures were grown in continuous substrate-limited mode, followed by transcriptome sequencing (RNA-Seq) of the total mRNA. Differential expression analysis showed that a cluster of genes surrounding the gene for TcDH were strongly upregulated during growth with thiocyanate. This cluster includes genes for putative copper uptake systems (*copCD*, ABC-type transporters), a putative electron acceptor (*fccAB*), and a two-component regulatory system (histidine kinase and a σ^54^-responsive Fis family transcriptional regulator). Additionally, we observed the increased expression of RuBisCO and some carboxysome shell genes involved in inorganic carbon fixation, as well as of *aprAB*, genes involved in sulfite oxidation through the reverse sulfidogenesis pathway.

**IMPORTANCE** Thiocyanate is a moderately toxic and chemically stable sulfur compound that is produced by both natural and industrial processes. Despite its significance as a pollutant, knowledge of the microbial degradation of thiocyanate is very limited. Therefore, investigation of thiocyanate oxidation in haloalkaliphiles such as the genus *Thioalkalivibrio* may lead to improved biotechnological applications in wastewater remediation.

## Abstract (precis)

Thiocyanate is a moderately toxic and chemically stable sulfur compound that is produced by both natural and industrial processes. Despite its significance as a pollutant, knowledge of the microbial degradation of thiocyanate is very limited. Therefore, investigation of thiocyanate oxidation in haloalkaliphiles such as the genus *Thioalkalivibrio* may lead to improved biotechnological applications in wastewater remediation.

## INTRODUCTION

Soda lakes are saline alkaline lakes found in (semi)arid regions around the world, such as the Altai Steppe in the Russian Federation, Mongolia, and northern China; the East African Rift Valley; Turkey; and parts of western North America (1). They are characterized by the presence of soluble sodium carbonate species (CO_3_^2−^ and HCO_3_^−^) at molar concentrations, which provides strong alkaline buffering that maintains a stable elevated pH, typically between 9 and 11. The total salinity of these lakes can rise because of evaporative concentration, sometimes up to saturation (>4.3 M Na^+^) (2).

Despite their haloalkaline character, soda lakes, even hypersaline ones, harbor a rich diversity of haloalkaliphilic prokaryotes and are extremely productive habitats with active biogeochemical cycles (3, 4). One of the most important of those is the sulfur cycle, whereby reduced sulfur compounds are oxidized by populations of both phototrophic and chemotrophic sulfur-oxidizing bacteria (SOB) and are recycled by sulfidogens (5–7). The dominant group of chemolithotrophic SOB found in soda lakes worldwide belongs to the genus *Thioalkalivibrio* (family *Ectothiorhodospiraceae*, class *Gammaproteobacteria*). They are obligate haloalkaliphiles with the ability to metabolize a diverse set of reduced sulfur compounds, including sulfide, polysulfide, elemental sulfur, thiosulfate, and tetrathionate, over a broad salinity range (8). Some strains have been shown to be capable of growth with thiocyanate as the sole electron donor and N source by using a pathway distinct from that used by characterized neutrophilic thiocyanate-oxidizing SOB (8–10).

Thiocyanate (N=C−S^−^) is a moderately toxic C_1_ sulfur compound that can be formed naturally, by the breakdown of glucosinolate compounds from plants or the detoxification of cyanide by rhodaneses, or in industrial processes, especially mining (11). Although there are several groups of bacteria that can utilize thiocyanate as a nitrogen source, only a small number of species can use it as an electron donor (12). Two pathways for the primary degradation of thiocyanate by SOB have previously been suggested (13). The carbonyl sulfide (COS) pathway, in which thiocyanate hydrolase cleaves the nitrile bond and produces COS as an intermediate, which is subsequently hydrolyzed to carbon dioxide and hydrogen sulfide by COS hydrolase. The existence of this pathway has been confirmed in neutrophilic *Thiobacillus* species that possess a cobalt-containing thiocyanate hydrolase, a nitrile hydratase homologue (14–16). The second suggested mechanism is the cyanate pathway, in which the C−S bond is hydrolyzed, producing cyanate and sulfide as the intermediates. However, the hydrolytic nature of this pathway has recently been called into question in a study of the mechanism of thiocyanate oxidation in haloalkaliphilic *Thioalkalivibrio* species (10, 17). It has been demonstrated that primary thiocyanate degradation in these SOB is only possible under aerobic conditions and that it results in the formation of elemental sulfur, rather than sulfide, in addition to cyanate. The enzyme responsible for the reaction is a periplasmic 56-kDa copper-containing oxidoreductase named thiocyanate dehydrogenase (TcDH). The presence of this gene has so far only been reported in the genomes of 10 *Thioalkalivibrio* species (18), as well as that of *Thiohalobacter thiocyanaticus* FOKN1 (19). Two structures of TcDH from two *Thioalkalivibrio* species have recently been made public in the Protein Data Bank (IDs 5F30 and 5F75), but the precise reaction mechanism has not yet been elucidated. Recently, we described the results of a comparative analysis of a cluster of genes surrounding the gene for TcDH that is found in 10 *Thioalkalivibrio* strains in two different gene configurations, although it remains unknown whether this cluster has an actual role in thiocyanate metabolism (18).

Here, we describe the results of a follow-up transcriptomics experiment comparing the growth of the thiocyanate-oxidizing strain *Thioalkalivibrio thiocyanoxidans* ARh 2^T^ with either thiosulfate or thiocyanate as an electron donor. The cultures were grown under tightly controlled conditions in substrate-limited chemostat mode to reduce differences in gene expression due to confounding factors, such as growth phase, whereby transcriptome sequencing (RNA-Seq) was used to quantify gene expression. The goal of this experiment was to identify genes whose expression increases specifically during growth with thiocyanate.

## RESULTS AND DISCUSSION

**FIG 1:**
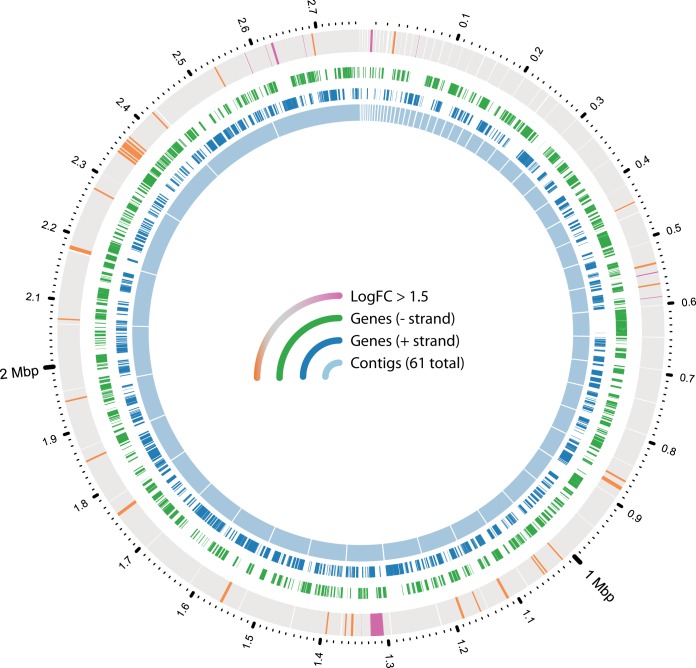
Overview of genes with high differential expression. The inner ring shows the layout of the genome of *T. thiocyanoxidans* ARh 2^T^ in 61 contigs. The two middle rings show the genes annotated on these contigs (blue, positive strand; green, negative strand). The outer ring shows genes with a logFC of >1.5 in color (purple, thiocyanate cultures; orange, thiosulfate cultures) and all other genes in gray.

To identify genes involved in the oxidation of thiocyanate, parallel chemostat cultures of *T. thiocyanoxidans* growing on either thiocyanate or thiosulfate as an electron donor were set up. At steady state, with an optical density at 600 nm (OD_600_) of 0.13 ± 0.01 for thiocyanate cultures and 0.34 ± 0.01 for thiosulfate cultures, biomass was harvested and its RNA was extracted and sequenced. The basic properties of the resulting RNA-Seq data are summarized in Table S1 in the supplemental material. On average, 8.5 million reads were produced per sample. A median of 21,280 rRNA reads were identified by SortMeRNA (20), with sample D being a strong outlier with 1.9 million reads. Additionally, a median of 628,828 reads were mapped to tmRNA, with an outlier in sample C-2 of 1.2 million. No trimming or filtering was performed because the quality of the reads was sufficient. Nearly all reads (98%) were successfully mapped to the reference genome, which is expected for a pure culture. Per sample, between 68 and 81% of the reads were unambiguously assigned to open reading frames (ORFs). Differential expression analysis of the read counts with DESeq2 yielded 101 ORFs with an absolute log_2_ fold change (logFC) of >1.5, an adjusted *P* value (*P*_adj_) of <10^−5^, and just under half (48) being annotated as encoding hypothetical proteins. Of the 101 strongly differentially regulated ORFs, 60 were upregulated during growth on thiocyanate, i.e., had a logFC of >1.5 (Table 1), and 41 were upregulated during growth on thiosulfate, i.e., had a logFC of ≤1.5. Figure 1 shows the small number of highly differentially expressed ORFs relative to the total number of genes. This clearly shows the power of using steady-state continuous cultures in comparative transcriptomics experiments, as noise usually resulting from cells being in different growth phases in batch cultures is eliminated. This justifies increased confidence in the conclusion that the differential expression observed in these cultures is indeed due to the different growth conditions and not due to other effects. However, it should be noted that the nitrogen contents of the two different chemostat conditions were partially different: 10 mM cyanate was formed as a result of thiocyanate oxidation, which would potentially form 10 mM ammonia by spontaneous hydrolytic degradation, which is twice the amount of ammonia present in thiosulfate-fed cultures. It was previously suggested that these bacteria lack, or suppress, cyanase activity (an enzyme hydrolyzing cyanate to ammonia and CO_2_) specifically to prevent the accumulation of toxic ammonia, using the relative stability of cyanate at elevated pH to their advantage (9, 10). Because of the high pH, the ammonia was mostly present as NH_3_ and would have been partially stripped from the culture by aeration. There are two systems for ammonium assimilation in bacteria: the glutamine synthetase/glutamine oxoglutarate aminotransferase (GS/GOGAT) system under low-ammonium conditions and the glutamate dehydrogenase (GDH) system when the ammonium concentration is high. Our data showed logFCs of −0.07 and −0.24 for the GS/GOGAT system (G372_RS0110205/G372_RS0113200) and 0.71 for GDH (G372_RS0107045). These changes are in line with the difference between the ammonium loads in the reactors.

10.1128/mSystems.00102-17.2 TABLE S1  Basic properties of the RNA-Seq data. An average of 8.5 million reads were produced per sample. Reads were mapped with tmap. A-1/A-2 and C-1/C-2 are technical replicates sequenced from the same biomass. Each count is the total number of reads that aligned unambiguously with an annotated feature in the genome. The percentage counted is the number of reads unambiguously aligned with annotated features divided by the total number of raw reads. Download TABLE S1, DOCX file, 0.02 MB. Copyright © 2017 Berben et al. 2017 Berben et al. This content is distributed under the terms of the Creative Commons Attribution 4.0 International license.

**TABLE 1:** Overview of the ORFs most strongly upregulated during growth with thiocyanate^a^

Locus tag	logFC (TC/TS)	*P* value	Annotation
G372_RS0100045	2.15	2.71E-15	GTP-binding protein TypA
G372_RS0100325	1.50	2.11E-8	Preprotein translocase subunit YajC
G372_RS0100555	1.58	6.88E-13	Hypothetical protein
G372_RS0100595	1.77	2.36E-12	30S ribosomal protein S12
G372_RS0100640	1.77	5.67E-8	tRNA-Trp
G372_RS0100755	1.82	2.25E-12	Hypothetical protein
G372_RS0100970	1.69	2.60E-16	50S ribosomal protein L3
G372_RS0100975	1.52	2.39E-11	30S ribosomal protein S10
G372_RS0101385	1.64	1.11E-5	Nucleoside triphosphate pyrophosphohydrolase
G372_RS0101425	1.76	7.96E-7	Glutaredoxin
G372_RS0101510	1.75	2.01E-12	30S ribosomal protein S6
G372_RS0101520	1.59	3.63E-12	Hypothetical protein
G372_RS0102735	1.73	2.80E-10	Sulfite oxidase
G372_RS0102740	1.52	6.26E-12	6,7-Dimethyl-8-ribityllumazine synthase
G372_RS0102750	1.70	7.83E-17	30S ribosomal protein S20
G372_RS0102895	1.93	1.20E-14	Hypothetical protein
G372_RS0102900	1.77	1.47E-10	Adenylyl-sulfate reductase subunit alpha
G372_RS0102905	1.65	3.73E-7	Adenylyl-sulfate reductase subunit beta
G372_RS0103005	1.86	3.75E-11	30S ribosomal protein S15
G372_RS0103030	2.37	2.73E-21	Ribosome maturation protein RimP
G372_RS0103115	2.07	5.26E-13	Preprotein translocase subunit SecG
G372_RS0103600	1.75	3.80E-13	Endonuclease YncB, thermonuclease family
G372_RS0104985	2.00	1.51E-16	Hypothetical protein
G372_RS0105180	3.45	3.98E-21	Hypothetical protein
G372_RS0105270	2.02	1.37E-18	Metal-binding protein
G372_RS0105600	1.74	2.04E-16	Hypothetical protein
G372_RS0105680	1.74	4.70E-12	NrdR family transcriptional regulator
G372_RS0106090	1.59	8.01E-16	Peptidyl-tRNA hydrolase
G372_RS0106285	3.00	4.61E-24	BNR repeat domain-containing protein
G372_RS0106290	3.64	1.32E-23	Iron outer membrane complex
G372_RS0106295	5.82	4.22E-21	Putative flavocytochrome *c*, cytochrome subunit
G372_RS0106300	6.94	2.09E-14	Putative flavocytochrome *c*, flavoprotein subunit
G372_RS0106305	4.01	2.03E-17	Putative TatA
G372_RS0106310	3.95	5.23E-15	Putative CopD
G372_RS0106315	4.74	1.21E-13	Putative CopC
G372_RS0106320	7.47	1.60E-17	Putative TcDH
G372_RS0106325	6.75	2.32E-18	Putative ABC-type transporter subunit
G372_RS0106330	5.31	1.02E-24	Putative ABC-type transporter subunit
G372_RS0106335	4.54	5.03E-26	Putative ABC-type transporter subunit
G372_RS0106340	5.19	7.36E-24	Putative ABC-type transporter subunit
G372_RS0106345	5.07	2.52E-24	Hypothetical protein
G372_RS0106350	3.97	7.64E-24	Histidine kinase
G372_RS0106355	2.02	2.33E-16	Fis family transcriptional regulator
G372_RS0106360	1.64	2.69E-11	Hypothetical protein
G372_RS0106445	2.58	1.65E-24	ATP-dependent RNA helicase DeaD
G372_RS0107190	2.15	1.34E-15	Guanylate kinase
G372_RS0107225	1.92	9.00E-8	50S ribosomal protein L33
G372_RS0108120	1.81	5.02E-22	Hypothetical protein
G372_RS0108320	2.05	1.30E-15	tRNA-Arg
G372_RS0108660	2.59	1.06E-10	tRNA-Gln
G372_RS0110325	1.52	1.26E-9	Membrane protein insertion efficiency factor
G372_RS0110505	1.76	2.82E-17	Endonuclease
G372_RS0112400	1.69	3.79E-15	Membrane protein
G372_RS0112490	1.54	7.29E-11	Inositol monophosphatase
G372_RS0112645	1.85	5.87E-17	Hypothetical protein
G372_RS0112650	1.67	2.50E-14	Hypothetical protein
G372_RS0112680	1.57	3.59E-6	Ribulose bisphosphate carboxylase
G372_RS0112685	2.07	4.32E-9	Ribulose bisphosphate carboxylase small chain
G372_RS0112690	1.52	2.93E-8	Carboxysome shell protein
G372_RS0112940	1.76	7.91E-14	Sulfate transporter

a Sixty ORFs showed a logFC of ≥1.5. For the complete data set (including ORFs upregulated during growth with thiosulfate), see Table S2. TC, thiocyanate; TS, thiosulfate.

To test the reliability of the differential expression data, we included one technical replicate, i.e., the same biomass sequenced twice, and three biological replicates, i.e., biomass harvested from three parallel cultures, for each electron donor. Analysis of the technical replicates revealed the absence of ORFs with a significantly different gene count (*P*_adj_ of >0.1 for all ORFs), which showed that there were no problems with library preparation or sequencing (data not shown). Figure S1 shows the first two principal components of a principal-component analysis of the biological replicates. The samples from the thiosulfate-fed reactors clustered together very closely on both axes, whereas the samples from the thiocyanate-fed cultures were more spread out along the second principal component (*y* axis). However, the second principal component represents only 12% of the variance in the data and the thiocyanate samples form a cluster that is clearly distinct from the thiosulfate-fed cultures, validating our analysis.

10.1128/mSystems.00102-17.1 FIG S1  Principal-component analysis of the variance among all RNA-Seq samples. The samples from thiosulfate-fed cultures cluster together very closely, whereas the samples from thiocyanate-fed cultures are spread farther apart. Download FIG S1, EPS file, 0.7 MB. Copyright © 2017 Berben et al. 2017 Berben et al. This content is distributed under the terms of the Creative Commons Attribution 4.0 International license.

### Thiocyanate metabolism.

**FIG 2:**
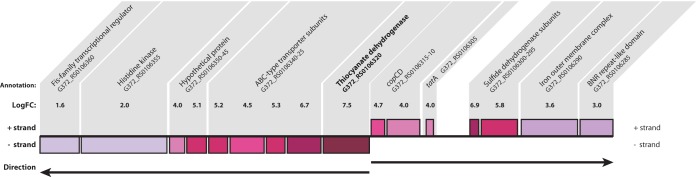
Changes in the expression of the gene for TcDH and neighboring genes. Of all of the ORFs in the transcriptome of *T. thiocyanoxidans*, the gene for TcDH shows the largest change during growth on thiocyanate, with a logFC of 7.5. Adjacent genes also show increased expression, although the effect is not as strong. The arrows indicate the transcriptional directions of the ORFs.

Previously, we described a cluster of genes associated with the TcDH-encoding gene that was found in two distinct genotypes in 10 *Thioalkalivibrio* strains (18). Differential expression analysis shows that when the culture is grown on thiocyanate, the gene for TcDH (G372_RS0106320) has the greatest change in expression compared to growth on thiosulfate (logFC = 7.5, *P*_adj_ = 1.6·× 10^−17^). Furthermore, the other genes in the previously described cluster show logFCs ranging from 1.6 to 6.9, with similarly low *P* values (Fig. 2).

The TcDH protein requires copper ions as cofactors for its activity, as shown in the structures published in the Protein Data Bank (PDB IDs 5F30 and 5F75). It seems likely that the *copCD* genes (C, G372_RS0106315; D, G372_RS0106310) located upstream from TcDH on the opposite strand are involved in the copper uptake process, especially considering *copC*’s 4.7-fold increase in expression. However, experimental determination of the exact mechanisms of copper acquisition by *T. thiocyanoxidans* and its incorporation in TcDH remains to be done. Located upstream from *copCD* is a *tatA* gene (G372_RS0106305) involved in the transport of folded proteins across the cell membrane whose expression was increased 4-fold. Two of the genes of interest in this cluster were predicted to contain *tat* signal peptides: TcDH itself and the flavoprotein subunit of *fcc* (21). The *tatA* gene is followed by two subunits of a flavocytochrome *c* sulfide dehydrogenase (*fcc*), the cytochrome subunit—which was upregulated 6.9-fold during growth with thiocyanate—and the flavoprotein subunit—which was upregulated 5.8-fold. However, unlike other Fcc sulfide dehydrogenases, such as that of *Allochromatium vinosum*, the cytochrome subunit contains a single heme binding site rather than two (22). In theory, the product of *fccAB* could function as a sulfide dehydrogenase, given that the active site is the flavin adenine dinucleotide-containing subunit, rather than the single heme cytochrome subunit. This was previously demonstrated for a monoheme *fcc* in *Thiobacillus* sp. strain W5 (23). However, no sulfide formation was observed in previous thiocyanate oxidation experiments with washed cells (10). We therefore speculate that its role may be to accept two electrons from TcDH during oxidation of the sulfane atom of thiocyanate to sulfur. Further biochemical research is necessary to confirm or refute this hypothesis.

**FIG 3:**
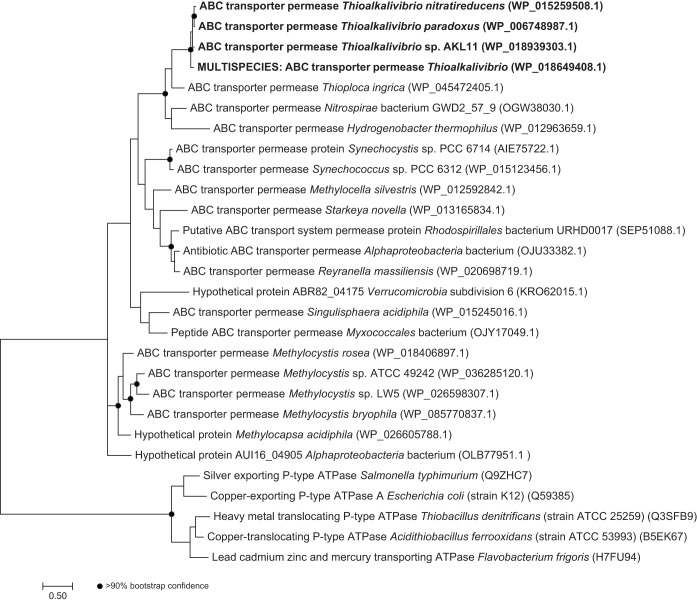
Maximum-likelihood phylogenetic tree, based on protein sequences, of an ABC permease (G372_RS0106325) found in the cluster of genes surrounding the gene for TcDH. Sequences found in TcDH-positive *Thioalkalivibrio* species (bold) cluster together. Black circles indicate nodes with >90% bootstrap confidence. Multispecies sequence record WP_018649408.1 represents identical sequences from the genomes of TcDH-positive *Thioalkalivibrio* sp. strains ARh 2/3/4/5, ALJ 4/5, and AL 5.

Directly downstream from the gene for TcDH are four genes coding for ABC-type transporter subunits, forming two pairs of permease/ATPase domains (G372_RS0106325/30 and G372_RS0106335/40). The increases in expression during growth on thiocyanate were similar for the ATPase subunits (5.2-fold and 5.3-fold), but the expression of the permease subunit encoded by G372_RS0106325 was increased 6.7-fold rather than the 4.5-fold increase in the expression of G372_RS0106335. The first hypothesis regarding the function of the ABC-type transporter genes is that they could be involved in copper transport into the cell. P_1B_-type ATPases are a relatively well-studied group of transporters, some of which are capable of copper translocation (24). In this family, a number of amino acid residues and motifs are conserved and required for copper transport. None of these features were found in the transporter genes present in the TcDH cluster in *T. thiocyanoxidans*: P_1B_-type ATPases have six to eight transmembrane helices, whereas the gene products in question were predicted to have only four by TMHMM (25). Additionally, no metal-binding domains were predicted by InterProScan (26). Figure 3 shows a phylogenetic tree of the permease G372_RS0106325 and related sequences as determined by a BLAST search against the RefSeq nonredundant (NR) protein database. Identical or highly similar sequences are found in all TcDH-containing *Thioalkalivibrio* genomes, and they all cluster together in the tree. These related sequences have the same transmembrane helix structure (one N-terminal helix, three C-terminal helices), except WP_018131508.1, which has an additional N-terminal helix; KRP34113.1, which has a fourth C-terminal helix; AIE75722.1, which lacks the N-terminal helix; and KRO62015.1, KRP32447.1, and KRP34113.1, which lack the N-terminal helix but contain a fourth C-terminal helix. P-type ATPase sequences were included in the tree as the outgroup. In contrast, a BLAST search of ABC permease G372_RS106335 against the NR protein database yielded hits only in TcDH-positive *Thioalkalivibrio* and in *Thioploca ingrica*. These data support the hypothesis that these genes have a specific function in thiocyanate metabolism, but its exact nature needs to be studied biochemically.

Downstream from the transporter genes, we found two ORFs annotated as hypothetical proteins (G372_RS0106345 and G372_RS0106350) whose expression increased 5.1-fold and 4.0-fold, respectively. Although no specific function prediction could be made for these genes, SignalP and TMHMM prediction showed a single N-terminal transmembrane helix in G372_RS0106345, which is therefore likely membrane anchored, and a putative *tat* signal in G372_RS010650, which is likely transported to the periplasm, where TcDH itself is located as well.

Last, downstream of these hypothetical proteins are located two genes forming a two-component regulatory system. G372_RS0106355 encodes a histidine kinase containing a GAF domain, and G372_RS0106360 encodes a σ^54^-specific Fis family transcriptional regulator. The increase in the expression of these genes is small compared to the rest of the putative TcDH operon, although the expression of a sensory system would not necessarily need to be increased upon the detection of its target. The transcriptional regulator is upregulated approximately 2-fold. The predicted protein sequence contains the GAFTGA motif that appears to be essential for its function as a σ^54^ enhancer-binding protein (EBP) (27). The genome of *T. thiocyanoxidans* ARh 2^T^ contains a single gene annotated as encoding an RNA polymerase σ^54^ factor (G372_RS0106205) whose expression does not change dramatically (0.4-fold up during growth on thiosulfate; *P* value of 0.02) and 11 putative σ^54^ EBPs, 8 of which contain the GAFTGA motif (Table 2). Only three of these putative EBPs showed a logFC of ≥1.5, i.e., G372_RS0106360, which presumably regulates the TcDH operon; G372_RS0111330 in the vicinity of an operon of genes annotated as encoding a PEP-CTERM domain-containing protein (downregulated during growth on thiocyanate); and G372_RS0112645 six genes upstream of RuBisCO. PEP-CTERM domain-containing proteins are currently thought to form a protein-sorting system in Gram-negative bacteria similar to the LPXTG/sortase system in Gram-positive bacteria and are associated with exopolysaccharide-producing enzymes (28). Their role in *Thioalkalivibrio* is unknown, but the expression data suggest that they have some role in thiosulfate metabolism. The upregulation of RuBisCO (see below) and a σ^54^ EBP in its vicinity suggest that σ^54^ may be a common regulatory actor in these processes.

**TABLE 2:** Putative σ^54^ EBPs^a^

Locus tag	Annotation	GAFTGA motif present	logFC	Genomic context
G372_RS0100690	Nitrogen regulation protein NR(I)	Yes	0.3	
G372_RS0104270	σ^54^-dependent Fis family transcriptional regulator	Yes	−1.2	
G372_RS0104475	σ^54^-dependent Fis family transcriptional regulator	Yes	−0.2	
G372_RS0104480	σ^54^-dependent Fis family transcriptional regulator	Yes	−0.4	
G372_RS0104625	σ^54^-dependent Fis family transcriptional regulator	No^b^	−0.4	
G372_RS0106360	σ^54^-dependent Fis family transcriptional regulator	Yes	2.0	TcDH operon
G372_RS0106870	σ^54^-dependent Fis family transcriptional regulator	Yes	−0.8	
G372_RS0109755	Nitrogen assimilation regulatory protein NtrX	No	−0.1	
G372_RS0111330	σ^54^-dependent Fis family transcriptional regulator	Yes	−1.6	Near operon of PEP-CTERM domain-containing proteins
G372_RS0112590	σ^54^-dependent Fis family transcriptional regulator	No^c^	−0.7	
G372_RS0112645	σ^54^-dependent Fis family transcriptional regulator	Yes	1.9	Six genes upstream from RuBisCO/carboxysome operon

a Eight of the 11 putative EBPs contain the essential GAFTGA motif. A negative logFC means that the gene was downregulated during growth with thiocyanate. Only three putative EBPs had an absolute logFC of ≥1.5; for these genes, the genomic context is included.

b GAFSGA.

c GAYTGA.

### Other genes of interest. (i) Inorganic carbon fixation.

RuBisCO is the key enzyme of the Calvin-Benson pathway that *Thioalkalivibrio* bacteria utilize for inorganic carbon fixation. It consists of large and small subunits, both of which were upregulated during growth with thiocyanate (large, G372_RS0112680, logFC of 1.6; small, G372_RS0112685, logFC of 2.1). Many *Thioalkalivibrio* species, including *T. thiocyanoxidans*, are capable of producing carboxysomes, bacterial microcompartments where CO_2_ is concentrated to compensate for RuBisCO’s low affinity for CO_2_ and to prevent unwanted oxygenation reactions (29). The carboxysome components are encoded by seven genes, of which only *csoS2* (G372_RS0112690) was strongly upregulated in thiocyanate cultures (logFC of 1.5). The carboxysome shell-associated carbonic anhydrase gene *csoS3* (G372_RS0112695), which encodes a subtype of β-carbonic anhydrases (30), was upregulated 1-fold, and the other five genes showed little change (absolute logFC of <1). One possible explanation is that cyanate—a nitrogen-containing intermediate product of thiocyanate oxidation by TcDH—acts as an inhibitor of the carbonic anhydrase, requiring a higher rate of expression of the corresponding genes to maintain the necessary rate of carbon fixation. Cyanate has been shown to inhibit β-carbonic anhydrases in a number of yeast species (31) and to cause increased RuBisCO expression in *Synechococcus elongatus* PCC 7942 (32, 33).

### (ii) Sulfite oxidation.

The final step in inorganic sulfur oxidation is the oxidation of sulfite (SO_3_^2−^) to sulfate (SO_4_^2−^). There are two pathways for this reaction: (i) direct transfer of two electrons to either cytochrome *c* (*sorAB*) (34, 35) or menaquinone (*soeABC*) (36) and (ii) an indirect “reverse sulfidogenesis” pathway involving adenosine phosphosulfate (APS) as an intermediate, catalyzed by APS reductase (*aprAB*) and sulfate adenylyl transferase (*sat*) (34, 37) that is present in strain ARh 2^T^. The *aprAB* genes were upregulated during growth with thiocyanate (A, G372_RS0102900, logFC of 1.8; B, G372_RS0102905, logFC of 1.6), although the expression of *sat* (G372_RS0102915) was more or less unchanged (logFC of −0.5). Assuming that the reaction pathway from sulfur to sulfite is the same under both conditions, the reason for this change in expression is unknown. Once again, it is possible that the presence of cyanate somehow influences the further upstream reactions of the sulfur-oxidizing pathway in *Thioalkalivibrio*.

### Conclusions.

The goal of this study was to discover which genes encode the proteins involved in thiocyanate metabolism in haloalkaliphilic SOB by using a comparative transcriptomics analysis of parallel chemostat cultures of *T. thiocyanoxidans* ARh 2^T^. The role of TcDH had previously been proven biochemically. However, we have demonstrated that a group of genes that surround the gene for TcDH, previously speculated to be involved in this process, do indeed show greater expression during growth with thiocyanate as an electron donor than during growth on thiosulfate. This group of genes includes not only the gene for TcDH but also a gene for a putative electron acceptor, possible copper uptake genes, and genes for transporters and a putative regulatory system. Additionally, expression changes were detected in two core metabolism gene systems, RuBisCO and *aprAB*.

Although there are still many open questions regarding the process of thiocyanate oxidation by TcDH—chiefly, the precise enzymatic reaction mechanism—all in all, these findings represent an important step toward a complete understanding of thiocyanate oxidation via the cyanate pathway in haloalkaliphilic SOB of the genus *Thioalkalivibrio*.

## MATERIALS AND METHODS

### Bacterial cultivation.

*T. thiocyanoxidans* ARh 2^T^ was obtained from the collection of D. Y. Sorokin, at the Delft University of Technology. The growth medium used throughout these experiments contained 0.6 M Na^+^ soda buffer at pH 9.8 (17.5 g ⋅ liter^−1^ Na_2_CO_3_, 13.9 g ⋅ liter^−1^ NaHCO_3_, 6.2 g ⋅ liter^−1^ NaCl, 1.0 g ⋅ liter^−1^ K_2_HPO_4_) supplemented with (separately sterilized) 0.2 g ⋅ liter^−1^ MgCl_2_ ⋅ 6H_2_O and 1 ml · liter^−1^ trace mineral solution (38) with the final CuCl_2_ ⋅ 2H_2_O concentration increased to 30 μg ⋅ liter^−1^. Either potassium thiocyanate at 10 mM (VWR) or sodium thiosulfate (Na_2_S_2_O_3_ ⋅ 5H_2_O) at 40 mM (Sigma-Aldrich) was used as an electron donor and sulfur source. For growth with thiosulfate, ammonium chloride (NH_4_Cl) at 5 mM was added as the nitrogen source, while for growth with thiocyanate, the ammonia was formed from thiocyanate.

Continuous cultivation was performed with the Multifors 2 bioreactor system (Infors HT, Switzerland) equipped with a total of six cultivation vessels with a working volume of 1 liter each. Dissolved oxygen and pH were monitored with online electrodes (Finesse [Switzerland] or Mettler-Toledo [Switzerland]) and the Iris control software (Infors HT, Switzerland). The culture was kept at 30°C, stirred at 300 rpm, and sparged with compressed air at 2 liters/min supplied with a mass flow controller (Vögtlin, Switzerland). Medium and waste vessels were connected aseptically with stainless steel connectors.

For both conditions, i.e., growth on thiosulfate and growth on thiocyanate, the cultures were run in three biological replicates. Additionally, one sample from each condition was sequenced twice as a technical replicate. The bioreactors were inoculated with 100 ml of *T. thiocyanoxidans* batch cultures grown with the corresponding substrate. An initial batch phase was used to accumulate biomass in the reactors. The excess elemental sulfur produced by *T. thiocyanoxidans* during growth on thiosulfate was removed by emptying the reactor into a sterile bottle, cleaning and resterilizing the reactor, and transferring the culture back. The feed and outflow were subsequently switched on. The dilution rate was set at 0.043 h^−1^ and periodically checked by timing the flow from a burette connected between the medium vessel and the feed pump. Growth was monitored by OD_600_ measurement (after removal of the elemental sulfur from thiosulfate-fed reactors), and the depletion of thiocyanate was confirmed by using ferric nitrate reagent (39).

### RNA-Seq.

The biomass was harvested from the bioreactors at steady state after five volume changes and collected in 50-ml Greiner tubes. These were immediately placed into a centrifuge rotor that was precooled to 4°C. The biomass was collected by centrifugation at 7,000 × *g* for 5 min at 4°C. The supernatant was discarded, and the pellet was immediately frozen in liquid nitrogen and stored at −80°C until further processing.

Frozen pellets were homogenized with a mortar and pestle and resuspended in QIAzol Lysis Reagent (Qiagen, Germany). Total RNA was isolated and purified with the RNeasy kit (Qiagen). The purification process included on-column DNase treatment with the RNase-free DNase kit (Qiagen). The final concentration was measured with a NanoDrop ND2000 (ThermoFisher Scientific, United States), and the integrity of the RNA was assessed on the 2200 TapeStation with Agilent RNA ScreenTapes (Agilent Technologies, The Netherlands). The Illumina Ribo-Zero rRNA removal kit for Gram-negative bacteria (Illumina, USA) was used to deplete the rRNA. Bar-coded RNA libraries were generated with the Ion Total RNA-Seq kit v2 and the Ion Xpress RNA-Seq barcoding kit by following the manufacturer’s instructions (ThermoFisher Scientific). The 2200 TapeStation was used with Agilent D1000 ScreenTapes (Agilent Technologies) to assess the size distribution and yield. Sequencing templates were prepared on the Ion Chef System with the Ion PI Hi-Q Chef kit (ThermoFisher Scientific). Finally, sequencing was performed on the Ion Proton platform with an Ion PI Chip v3 (ThermoFisher Scientific) in accordance with the manufacturer’s instructions.

### RNA-Seq data analysis.

The genome of *T. thiocyanoxidans* ARh 2^T^ (GenBank accession no. NZ_ARQK00000000.1) was previously sequenced and annotated (40). The FASTA file containing the genome sequences (in a total of 61 contigs) and the GFF file containing all of the sequence annotations were downloaded from the NCBI RefSeq FTP server. The RNA-Seq reads were mapped to the reference genome with tmap 4.2.18 (ThermoFisher Scientific), and raw read counts were produced by HTseq (http://htseq.readthedocs.io/en/release_0.9.1/). Differential expression analysis was performed with DESeq2 version 1.14.1 (41), provided by the Bioconductor framework (42), after collapsing the read counts for the technical replicates. DESeq2 normalizes raw read counts by estimating size factors by a median-of-ratios method (43). The variability between replicates and noise due to ORFs with low total read counts are then reduced by empirical Bayes shrinkage methods (41). DESeq2 then tests the significance of the logFC estimate by using the Wald test with the *P* value adjusted for multiple testing (44). For the complete differential expression data, including NCBI locus tags, logFCs, *P*_adj_ values, raw read counts, and gene annotations, see Table S2. KEGG ortholog annotations were obtained by running the protein FASTA file obtained from the NCBI server through BlastKOALA (45) and merging the annotations with the differential expression table. Gene expression was visualized with Circos 0.69 (46). All logFCs were calculated as the log_2_ of the ratio of the read counts in thiocyanate cultures to the read counts in thiosulfate cultures. Therefore, a positive logFC means that an ORF was expressed more during growth with thiocyanate and a negative logFC means that a gene was expressed less during growth with thiocyanate.

10.1128/mSystems.00102-17.3 TABLE S2  Complete differential expression data. *T. thiocyanoxidans* ARh 2^T^ ORFs are listed with their logFCs, log counts per million, likelihood ratios, *P*_adj_ values, raw read counts per sample, and annotations. Download TABLE S2, XLSX file, 0.3 MB. Copyright © 2017 Berben et al. 2017 Berben et al. This content is distributed under the terms of the Creative Commons Attribution 4.0 International license.

### Phylogenetic analysis.

The phylogenetic tree in Fig. 3 was generated as follows. A protein BLAST search of G372_RS0106325 against the NR protein database was used to find similar sequences, which were subsequently aligned with each other by using Clustal Omega (47). Prottest 3 (48) was used to determine the optimal amino acid substitution model, i.e., LG gamma distributed (five discrete categories) with invariant sites (49). The maximum-likelihood tree was calculated with MEGA 7 (50) by using 500 bootstrap replicates.

### Accession number(s).

The raw RNA-Seq data obtained in this study have been deposited in the NCBI Sequence Read Archive under SRA accession numbers SRX3442449 to SRX3442456.
