# Supplementary material for: Analysis of the Genes Involved in Thiocyanate Oxidation during Growth in Continuous Culture of the Haloalkaliphilic Sulfur-Oxidizing Bacterium Thioalkalivibrio thiocyanoxidans ARh 2T Using Transcriptomics
Source: mSystems. 2017 Dec 26;2(6):e00102-17. doi: 10.1128/mSystems.00102-17 (PMC5744179; doi:10.1128/mSystems.00102-17)
Supplement: TABLE S1 [file sys006172159st1.docx]

**Supplementary Table S1: Basic properties of the RNA-seq data.** An average of 8.5 million reads was produced per sample. Reads were mapped using tmap. A-1/A-2 and C-1/C-2: technical replicates sequenced from the same biomass. Counts: sum of the number of reads that aligned unambiguously to an annotated feature in the genome. Percentage counted: number of reads unambiguously aligned to annotated features divided by the total number of raw reads.

| **Reactor** | **e^-^-donor** | **Total reads** | **Mapped reads** | **% mapped** | **Counts** | **% counted (of total reads)** | **No. rRNA reads** | **No. tmRNA reads** |
| --- | --- | --- | --- | --- | --- | --- | --- | --- |
| A-1 | SCN^-^ | 9,240,175 | 9,068,650 | 98 | 7,252,851 | 78 | 17,755 | 621,304 |
| A-2 | SCN^-^ | 8,740,675 | 8,602,420 | 98 | 6,558,663 | 75 | 21,104 | 560,231 |
| B | SCN^-^ | 9,380,308 | 9,233,229 | 98 | 7,062,236 | 75 | 19,459 | 678,818 |
| C-1 | S_2_O_3_^2-^ | 8,716,413 | 8,551,185 | 98 | 7,053,589 | 81 | 15,466 | 423,026 |
| C-2 | S_2_O_3_^2-^ | 8,360,801 | 8,195,087 | 98 | 5,720,332 | 68 | 25,654 | 1,218,024 |
| D | SCN^-^ | 8,763,763 | 8,645,602 | 98 | 7,066,651 | 81 | 1,925,455 | 492,702 |
| E | S_2_O_3_^2-^ | 7,103,237 | 6,997,740 | 98 | 5,323,025 | 75 | 23,844 | 636,352 |
| F | S_2_O_3_^2-^ | 7,707,199 | 7,571,847 | 98 | 5,481,802 | 71 | 21,312 | 936,643 |
